# Understanding Aromaticity in [5]Helicene-Bridged Cyclophanes: A Comprehensive Study

**DOI:** 10.1021/acs.joc.3c02485

**Published:** 2024-01-18

**Authors:** Mesías Orozco-Ic, Luis Soriano-Agueda, Sílvia Escayola, Dage Sundholm, Gabriel Merino, Eduard Matito

**Affiliations:** † Donostia International Physics Center (DIPC), Donostia, 20018 Euskadi, Spain; ‡ Institut de Química Computacional i Catàlisi and Departament de Química, 226245 Universitat de Girona, C/Maria Aurèlia Capmany, 69, Girona, 17003 Catalonia, Spain; § Department of Chemistry, Faculty of Science, University of Helsinki, A. I. Virtasen aukio 1, P.O. Box 55, FIN-00014 Helsinki, Finland; ∥ Departamento de Física Aplicada, Centro de Investigación y de Estudios Avanzados, Unidad Mérida, Km 6 Antigua Carretera a Progreso. Apdo. Postal 73, Cordemex, 97310 Mérida, Yuc., México

## Abstract

This study explores the aromaticity of doubly [5]­helicene-bridged (1,4)­cyclophane and triply [5]­helicene-bridged (1,3,5)­cyclophane via calculations of the magnetic response and of electronic aromaticity indices. The primary objective is to assess the π-electron delocalization to determine whether they sustain global ring currents associated with π aromaticity. The molecules show local ring currents in the presence of an external magnetic field. The ring currents flow diatropically in the stacked six-membered rings and in the helicene arms. However, these π currents are not interconnected due to the discontinuity of the π delocalization at the C–C single bonds connecting the central six-membered rings to the helicene arms. Electronic indices suggest that the helicene-arm systems have significantly smaller electron delocalization than benzene. The reduction in the delocalization does not compromise their ability to exhibit ring currents in the presence of an external magnetic field. The analysis provides further evidence that the magnetic criteria yield a different degree of aromaticity for the helicene arms than obtained in the calculation of the electronic aromaticity indices. However, both approaches confirm that the studied molecules are not globally aromatic.

## Graphical abstract

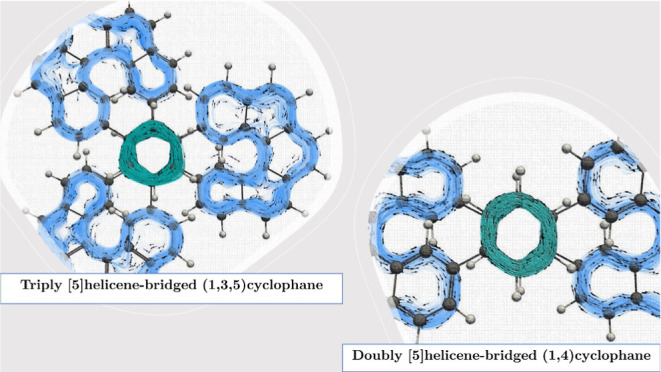

## Introduction

Recently, Kubo et al. reported the synthesis of an eight-shaped molecule (**I**, Figure 1) composed of two [5]­helicenes connected to two stacked six-membered rings (6-MRs), resulting in a doubly [5]­helicene-bridged (1,4)­cyclophane.^1^ In a more recent paper by Aribot et al.,^2^ they reported a structurally similar propeller-shaped molecule (**II**), consisting of three [5]­helicenes joined at their ends by two stacked 6-MRs. The helicene arms and the central stacked 6-MR are linked by three bridges consisting of C–C single bonds. Both molecules can be viewed as [2,2]­paracyclophanes, but instead of being connected by C–C bridges, they are linked by helicenes (see Figure 1). These kinds of molecules with chiral topologies are particularly appealing due to their exceptional optical properties, which can potentially be used in electronic applications.^1–7^ The aromatic nature is another interesting property of these molecules,^8,9^ as the electron delocalization along the nonplanar structures can significantly contribute to their stability.^10^

**Figure 1:**
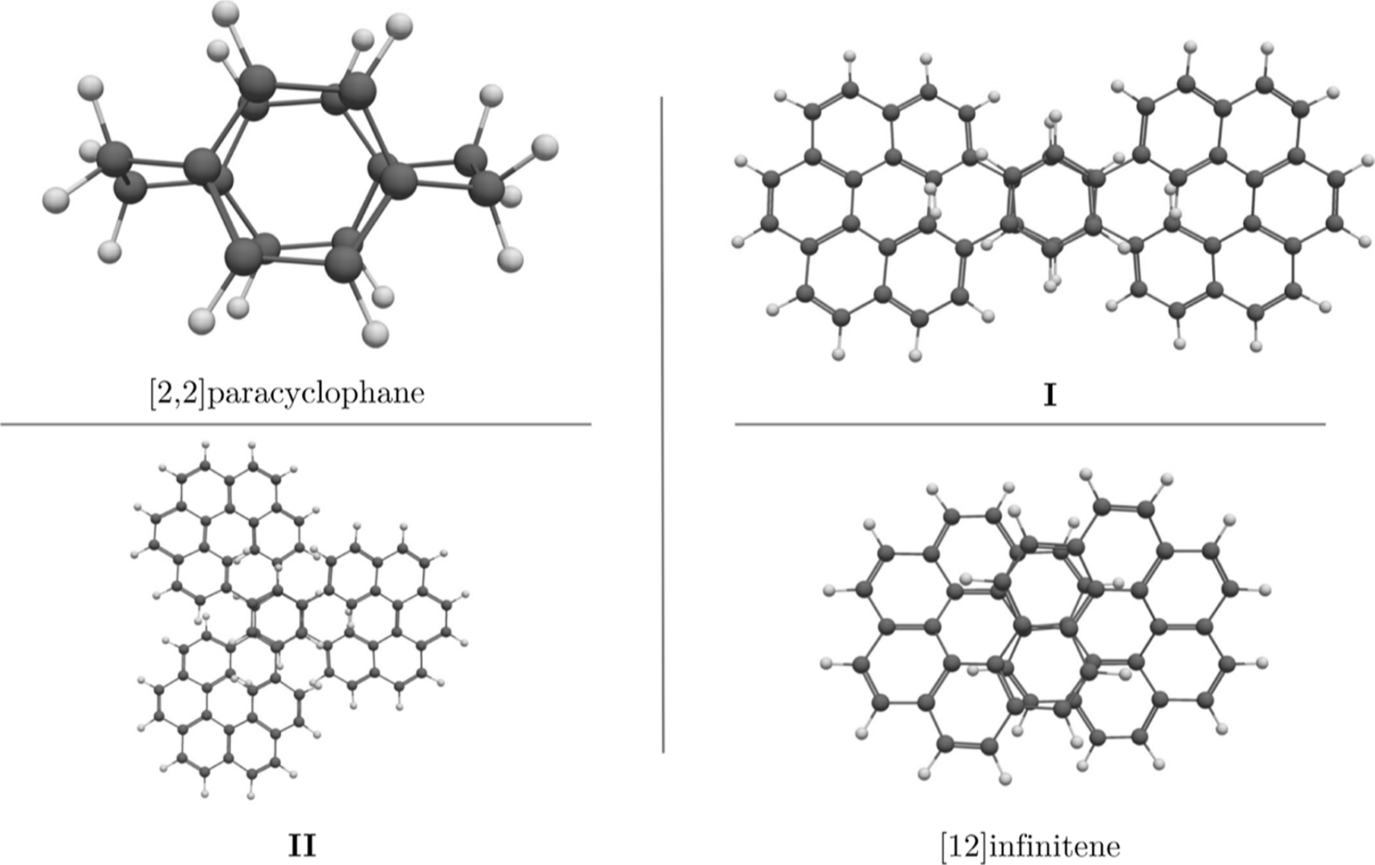
Molecular structures of [2,2]­paracyclophane, doubly [5]­helicene-bridged (1,4)­cyclophane (**I**), triply [5]­helicene-bridged (1,3,5)­cyclophane (**II**), and [12]­infinitene.

In the study by Aribot et al.,^2^ the aromaticity of the triply [5]­helicene-bridged (1,3,5)­cyclophane was investigated by performing calculations of the magnetic response and using the electron density of delocalized bonds^11^ (EDDB) function approach. The authors claimed that **II** exhibits a diatropic global 78 π-electron current–density response to an external magnetic field. They suggested that a ring current flows along the molecular frame, passing through the central rings and splits into the three helicene arms.^2^ They argued that the EDDB isosurfaces provide evidence of significant delocalization through the single C–C bonds linking helicene branches to the central 6-MRs. To support the obtained results, the authors performed calculations on (1,3,5)-triphenylbenzene-based models to elucidate the specific magnetic shieldings in **II**.

The aromaticity of three-dimensional systems, especially in helicenes, has proven to be challenging and controversial, particularly when using magnetic response analyses.^12,13^ Helical structures consist of stacked rings, which can complicate the interpretation of magnetic shielding calculations.^13–15^ It has been found that local nucleus-independent chemical shift^16,17^ (NICS) calculations assign different degrees of aromaticity to the individual rings of helicenes,^12,13^ which originates from the overlap of the shielding cones of the stacked rings. A more precise comprehension of the π delocalization has been obtained by performing an orbital analysis of the magnetic response.^13,18^ NICS(0) values are substantially affected by contributions parallel to the plane due to the orientation of the rings with respect to the external magnetic field.^19^ Interpretations based on these local magnetic indices may be misleading and an extensive analysis combining current–density and magnetic shielding calculations is better suited for systems with polycyclic or heteroatomic rings.^20–22^ For planar (and nonplanar) polycyclic systems, the shielding cones from neighboring rings may overlap making interpretations based on magnetic shielding calculations challenging.^18^ For example, the multipath character in the current density of large acenes complicates the determination and quantification of local (and global) aromaticity.^23–26^ Calculations involving the π-electron component of the magnetic response suggested that helicenes are globally aromatic since they sustain a global diatropic (clockwise) ring current along their perimeter,^18,27,28^ promoting the formation of a deshielding cone along the helical axis. The deshielding cone becomes more pronounced with increasing helicene size.^13,18^ Thus, no local ring currents flow around any of the helicene’s 6-MRs, making it inappropriate to characterize one ring as more or less aromatic than another. Furthermore, the integration of the global current density in helicenes shows that its degree of aromaticity is weaker as compared to benzene.^18,27^

An intriguing example of aromaticity within helicene-based systems can be found in [12]­infinitene, which consists of two [6]­helicenes fused at their ends, forming a doubly twisted eight-shaped Möbius strip with a crossing point featuring stacked rings.^3^ Unlike the molecules studied in this work, [12]­infinitene lacks C–C single-bond bridges. Instead, it exhibits two global π ring currents flowing along its edges, which do not intersect.^14,29^ Remarkably, [12]­infinitene fulfills the rule of cylindrical aromaticity. [12]­Infinitene is an exciting molecule that underscores the importance of considering the π contribution to the current density when evaluating the π-electron flux in three-dimensional molecules. This analysis aids in determining whether a molecule possesses global π aromaticity. Given that the **I** and **II** structures share a helicene-based framework and are connected by C–C single bonds at the stacked 6-MRs, it becomes pertinent to clarify whether these molecules exhibit global aromaticity. Unlike other globally aromatic systems,^10^ there is a discontinuity in their EDDB isosurfaces.^2^ However, the existence of C–C single bonds does not necessarily imply an interruption of the current–density flux.^30^ For instance, charged species of cycloparaphenylenes have been reported, in which C–C single bonds connect their 6-MRs, yet this does not prevent the existence of a global ring current,^31,32^ and global aromaticity. The π-electron magnetic behavior of **I** and **II** has not yet been resolved and their global aromatic character is elucidated in this work.

In this study, we investigate the aromaticity of **I** and **II** by analyzing the magnetically induced current density, induced magnetic field, and NICS values. Additionally, we compute various indices, including the delocalization index (DI) and electronic aromaticity indices (BOA,^33^ AV_min_,^34^ and AV1245 ^35^), along with the EDDB function to estimate the electron delocalization. The primary focus is on evaluating π-electron aromaticity. Our findings reveal that both **I** and **II** exhibit local π ring currents as the response to an external magnetic field. The ring currents circulate diatropically within the stacked 6-MRs and in the helicene-arms. However, we note that these π ring currents remain independent due to a disruption in π delocalization caused by the C–C bridges that connect the central rings to the helicene arms. Consequently, neither **I** nor **II** displays a global aromatic π character.

## Computational Details

All geometries were fully optimized using the CAM-B3LYP^36^ functional, in combination with the def2-TZVP^37^ basis set, and Grimme’s dispersion correction with Beck–Johnson damping D3­(BJ).^38^ Vibrational analysis was carried out to verify that the optimized structures are local minima on the corresponding potential energy surfaces. Cartesian coordinates of the optimized structures can be found in the Supporting Information. These calculations were performed with the Turbomole software.^39^ Given that hybrid long-range corrected functionals like CAM-B3LYP are well suited for describing electron delocalization in large organic molecules^40,41^ and provide magnetic properties consistent with those obtained at the CCSD­(T) level,^42^ we computed the electronic indices and the magnetic response at the CAM-B3LYP/def2-TZVP level. The magnetic response was determined by computing the magnetically induced current density^43–45^ (**J** ^ind^) and the induced magnetic field^46–48^ (**B** ^ind^) using the gauge including magnetically induced current (GIMIC)^43–45^ and Aromagnetic^49^ programs, respectively.

Gauge-including atomic orbitals^50,51^ (GIAOs) were employed for computing the magnetic properties using their interface to the Turbomole program. An external unit field (|**B** ^ext^| = 1 T) aligned along the *z*-axis, which is the highest molecular symmetry axis, was applied. **B** ^ind^ is mathematically defined as the matrix product of the magnetic shielding tensor by the external magnetic field.^48^ NICS values can be understood as the isotropic average strength of **B** ^ind^ using an external magnetic field in the three Cartesian directions. Under these circumstances, the *z*-component of **B** ^ind^ (*B*_*z*_^ind^) is the largest one, effectively reducing the **B** ^ind^ analysis to an examination of *B*_*z*_^ind^, which is equivalent to the *zz*-component of the NICS values (NICS_*zz*_).^16,17,52^ Thus, *B*_*z*_^ind^ is approximately one-third of the NICS for long distances over the molecular plane. GIMIC was used to calculate **J** ^ind^ plots and ring-current strengths by integrating the current density flowing through a plane intersecting one or more chemical bonds,^43–45^ which is a standard method for quantifying aromaticity. The units for the ring-current strengths are nA/T, while the NICS and *B*_*z*_^ind^ values are given in ppm.

For organic and all-carbon molecules with hybridization closely approximating sp^2^, it is possible to accurately estimate the magnetic response corresponding to the π-electron contribution via the pseudo-π model.^18,53–55^ The pseudo-π model involves computing the magnetic response of an optimized organic system with hydrogen atoms removed (if any), and the carbon centers replaced by hydrogen atoms at the same positions. Hence, we computed the induced current density (^pπ^ **J** ^ind^) and the induced magnetic field (^pπ^ **B** ^ind^) of the pseudo-π model.

The electronic aromaticity indices (BOA,^33^ AV_min_,^34^ and AV1245 ^35^) were computed using the quantum theory of atoms in molecules^56,57^ (QTAIM) atomic partition facilitated by the AIMAll software.^58^ The atomic overlap matrices resulting from this partition, in combination with wave functions from Turbomole, provided the input for the ESI-3D code,^33,59^ yielding numerical values for these indices. The error in the number of electrons was less than 0.01 au, and the maximum integrated Laplacian in the atomic domain was 10^–4^ au EDDB^11,60^ functions were also calculated.

We performed calculations of the EDDB functions that divide the one-electron density into several “layers” representing various levels of electron delocalization. The EDDB function reflects the population of delocalized electrons across all conjugated bonds in a molecule.^11,60^ We evaluated the EDDB_H_(r) function, that is, by excluding hydrogen atoms, which is recommended for organic molecules.^60^ The EDDB_H_(r) function along with its π-electron contribution (π-EDDB) provides insights to organic systems.^60^ The EDDB analysis, implemented in RunEDDB program, requires the one-electron density matrix that we calculated at the CAM-B3LYP/def2-TZVP level using the Gaussian 16 software,^61^ in conjunction with the natural bond orbital (NBO) version 3 code.^62^

## Results and Discussion

Let us start our discussion with an examination of the magnetic shielding properties of **I** (Figure 2). It became evident that the NICS and *B*_*z*_^ind^ isosurfaces yield completely different shielding behaviors. The NICS function has been commonly used to discuss aromaticity in three-dimensional systems because, unlike *B*_*z*_^ind^, it is a quantity that does not depend on the orientation of the external magnetic field. However, for helical structures, interpretations of aromaticity based on magnetic shielding functions differ from the insights gained through current–density computations.^13–15,27^ Namely, *B*_*z*_^ind^ displays a deshielding cone, which is typical for antiaromatic systems, whereas the NICS calculations predict an aromatic character, which is evident through the formation of shielding cones at all 6-MRs. The differences originate due to the helical structure, in which the induced current density flows along the perimeter of the helicenes.^13,18^ It is these changes in the direction of the current–density flux in conjunction with the orientation of the rings with respect to the external field that causes the in-plane *x*- and *y*-components of **B** ^ind^ to become important.^13,19^ Therefore, NICS(0) and *B*_*z*_^ind^(0) calculations provide a different aromatic nature for each ring of the studied molecules. When two stacked aromatic molecules such as the benzene dimer are considered, the overlap of their shielding cones is constructive and results in a more pronounced shielding.^12,13,63,64^ In the case of helical molecules, the overlap leads to a wrong interpretation.^13–15^ Our experience is that when different features are predicted by the NICS and *B*_*z*_^ind^ functions, it is often due to complex current–density pathways.^13–15^ Furthermore, the pseudo-π model, which is designed to mimic the magnetic response of the π electrons, yields a similar behavior for the NICS and *B*_*z*_^ind^ functions implying that characterizing the aromaticity of **I** based on magnetic shielding calculations is challenging.

**Figure 2:**
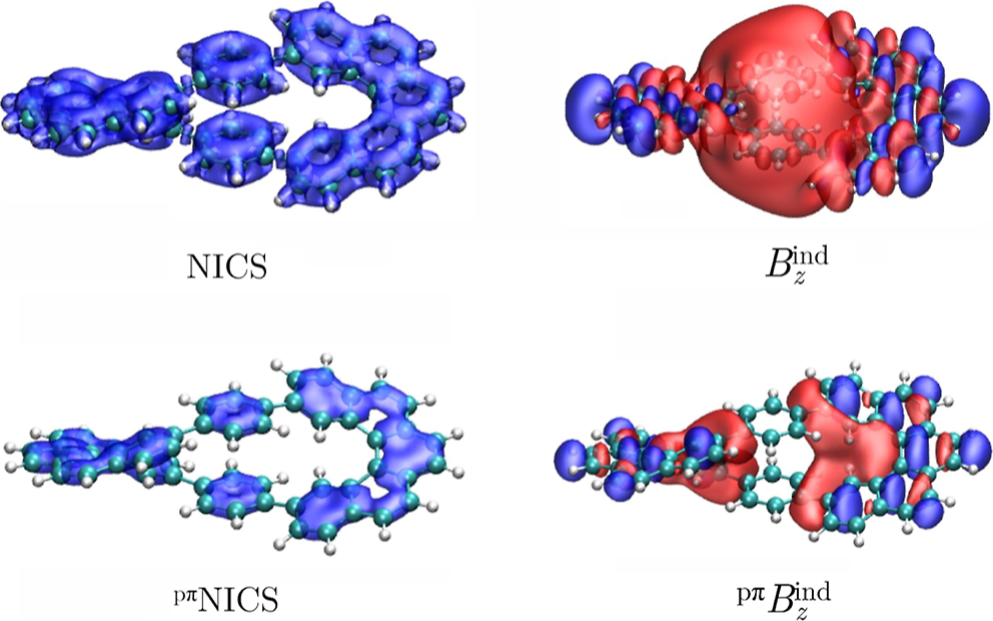
Isosurfaces of the total and pseudo-π modeled NICS and *B*_*z*_^ind^ for **I**. The shielding (−15 ppm) and deshielding (+15 ppm) cones are shown in blue and red, respectively.

The current–density calculations unveil a global diatropic circulation surrounding the perimeters of the helicene arms, which is connected to a local circulation in the central 6-MRs (see Figure 3). However, **J** ^ind^ computations encompass contributions from all electrons (core + σ + π), making it difficult to identify the π current–density pathways. Conversely, the ^pπ^ **J** ^ind^ plots indicate that **I** exhibits three different diatropic ring currents in the helicene branches, which formally consist of a pathway with twenty-two electrons in the bond conjugation (22-MR) along with local aromatic 6-MRs sustaining diatropic ring currents in the π orbitals (Figure 3). An interactive visualization of the magnetic response is provided through the current–density animations in the Supporting Information. Hence, there is no global current density in the π orbitals of **I**. Instead, the ring current consists of local 6-MR and 22-MR diatropic circulations. Integration of **J** ^ind^ results in a total (and a pseudo-π) ring-current strength of 11.67 (12.56) nA/T for the helicene-like pathways, while a ring-current strength of 10.11 (8.74) nA/T is obtained for the local circulations in the central 6-MRs (Table 1), implying that there is a local aromatic character in each of the helicene branches as well as in the central 6-MRs.

**Figure 3:**
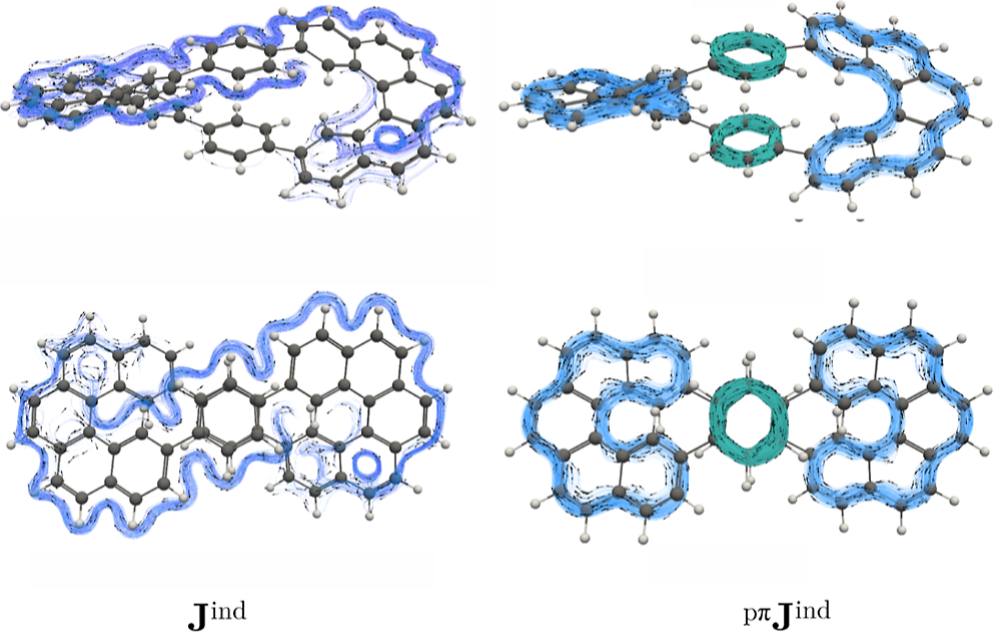
Streamlines and vector representation of the total and the pseudo-π modeled ring current density of **I**. Side view (top) and top view (bottom). The diatropic ring currents in the helicene branches and in the central 6-MR are shown in the ^pπ^ **J** ^ind^ in blue and green, respectively.

**Table 1:** Total and the Pseudo-π Ring-Current Strengths (in nA/T) for **I** and **II** are Divided Into the Diatropic and Paratropic Components Calculated at the CAM-B3LYP/def2-TZVP Level^a^

		total			pseudo-π		
molecule	plane	diatropic	paratropic	net	diatropic	paratropic	net
**I**	A	15.31	–5.20	10.11	8.79	–0.05	8.74
	B	16.49	–4.82	11.67	12.56	0.00	12.56
**II**	A	14.12	–5.86	8.26	7.56	–0.04	7.52
	B	16.57	–4.76	11.81	12.68	–0.01	12.67

a The values for planes A and B correspond to the ring-current strengths in the 6-MR and 22-MR (helicene-like), respectively. The integration planes are shown in Figure S1 of the Supporting Information.

According to the EDDB analysis, delocalized pathways in organic systems typically display continuous EDDB surfaces along carbon chains or around rings^11,60^ as for the 6-MRs of **I**. In the all-electron (total) EDDB calculations, the delocalization surfaces extend over the entire carbon skeleton, with a discontinuity at the C–C single-bond bridges that connect the helicene arms to the central 6-MRs (Figure 4). However, when considering only the π-electron contribution to the EDDB function (π-EDDB), there is a discontinuity at these C–C bonds as well as along a part of the outer edge of the 6-MRs of the helicene arms (Figure 4). Thus, the π-EDDB calculations also suggest that there is no global aromaticity in **I**. Furthermore, the EDDB analysis enables us to estimate the number of delocalized electrons in the different circuits. For example, each of the central 6-MRs is predicted to have about delocalized 4.83*e* according to the total EDDB­(r) function, while the π-EDDB partition suggests 4.60*e*. In the helicene-like circuit, the total and the π-component of the EDDB suggest 15.71 and 14.23 delocalized electrons, respectively (Table 2). These values seem to be counterintuitive, as one might expect π-EDDB values of about 6*e* (rather than 5*e*) in the 6-MRs due to the presence of three π orbitals on those rings. A similar exercise can be applied to the helicene-like circuit, leading to a value around 22*e*. However, this can be explained by the EDDB definition,^11,60^ which considers that the π-electron density population comprises π electrons localized on atoms (inner shells, lone pairs) (π-EDLA), π electrons localized between pairs (π-EDLB), and π electrons delocalized in conjugated bonds (π-EDDB).^65,66^ From this perspective, only 75 and 65% of the π-density are *multicentrically* delocalized around the 6-MRs and 22-MRs, respectively. The remaining share is attributed to π-EDLB + π-EDLA. For benzene, the π-EDDB component leads to 5.33*e* of delocalization (88%), implying that the π electrons of the 6-MRS are less delocalized than for benzene. From a global point of view, 40.18*e* from π-EDDB are delocalized in the whole molecule, corresponding to 72% of them.

**Figure 4:**
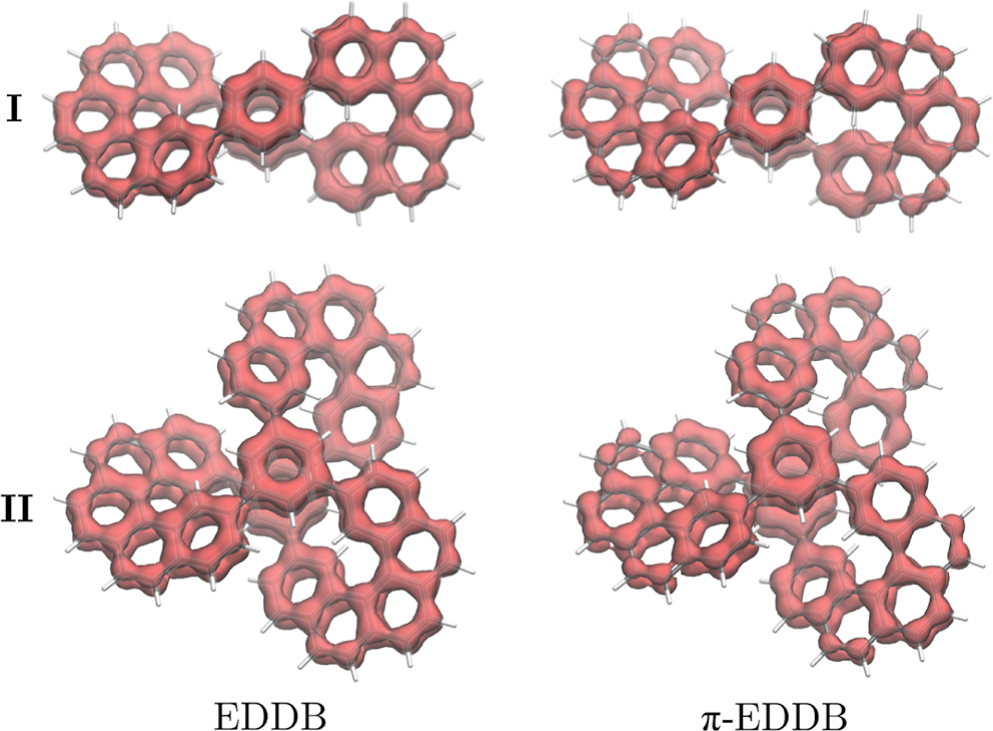
Total and the π contributions to the EDDB function are plotted for **I** and **II**. The plotted isosurfaces correspond to an isovalue of 0.02.

**Table 2:** Total and the π-Component EDDB Values (in *e* Units) for the 6-MR and 22-MR (Helicene-like) in **I** and **II** Computed at the CAM-B3LYP/def2-TZVP Level

molecule	ring	EDDB	π-EDDB
**I**	6-MR	4.83	4.60
	22-MR	15.71	14.23
	global	44.31	40.18
**II**	6-MR	4.77	4.53
	22-MR	15.69	14.23
	global	61.66	55.62

Another approach to address the presence of a delocalized global pathway involves calculating electronic indices. The delocalization index (DI) plays a crucial role as it quantifies the number of electrons that are delocalized or shared between two different atoms. However, when we apply DI to the C–C single-bond bridges connecting the branches with the central 6-MRs, we obtain a value of 1.05. This is a small value in comparison to a truly π-delocalized bond. For example, in benzene, the DI index in one of its C–C bonds is 1.40, of which approximately 1.0 originates from the σ electrons, while 0.4 comes from π electrons.^67^ Thus, there is a significant decrease in the π-contribution in the C–C bridges, suggesting that the delocalization in the C–C bridges originates only from the σ orbitals.

Furthermore, we have also examined the electron-delocalization pathways around the central 6-MRs and the 22-MRs by calculating the BOA, AV1245, and AV_min_ indices, whose values are reported in Table 3. These indices were selected because they can be applied to large rings.^68–70^ The BOA index has values close to zero (exactly zero for the central 6-MR), which is characteristic of aromatic rings like benzene with homogeneous DI values along the pathway. In contrast, the AV1245 and AV_min_ calculations yield large values for the 6-MRs indicating an aromatic character, while for the 22-MR pathway, the values are significantly smaller suggesting a weaker delocalization. Thus, when looking at these indices, it is evident that these indices do not indicate an aromatic character for the helicene-like arms. The difference is more discernible when observing the color paths in the AV1245 plots (see Figure S2) illustrating that the 6-MRs are substantially delocalized. In contrast, the 22-MRs have different degrees of delocalization along the perimeter, with the inner edge displaying the least delocalization. Globally, the BOA values are larger than for the individual 6-MR and 22-MR pathways, while the AV1245 values are similar to those of the 22-MR. Hence, **I** is not globally aromatic according to the electronic indices as well.

**Table 3:** Electronic Indices (BOA, AV1245, AV_min_) for the 6-MR, 22-MR (Helicene-like), and for the Whole Structure of **I** and **II**, Computed at the CAM-B3LYP/def2-TZVP Level

molecule	ring	BOA	AV1245	AV_min_
**I**	6-MR	0.0513	8.41	7.87
	22-MR	0.1943	2.52	0.13
	global	0.2236	1.31	0.18
**II**	6-MR	0.0178	7.64	7.64
	22-MR	0.1943	2.51	0.13
	global	0.2196	1.45	–0.02

The magnetic response of **II** is very similar to the one of **I**. The NICS isosurfaces exhibit shielding cones throughout the structure with fused cones at the helicene arms, while the central 6-MRs have smaller individual shielding cones. The magnetic response obtained in the *B*_*z*_^ind^ calculations is paramagnetic at the center of the molecule indicating an antiaromatic character (Figure 5).

**Figure 5:**
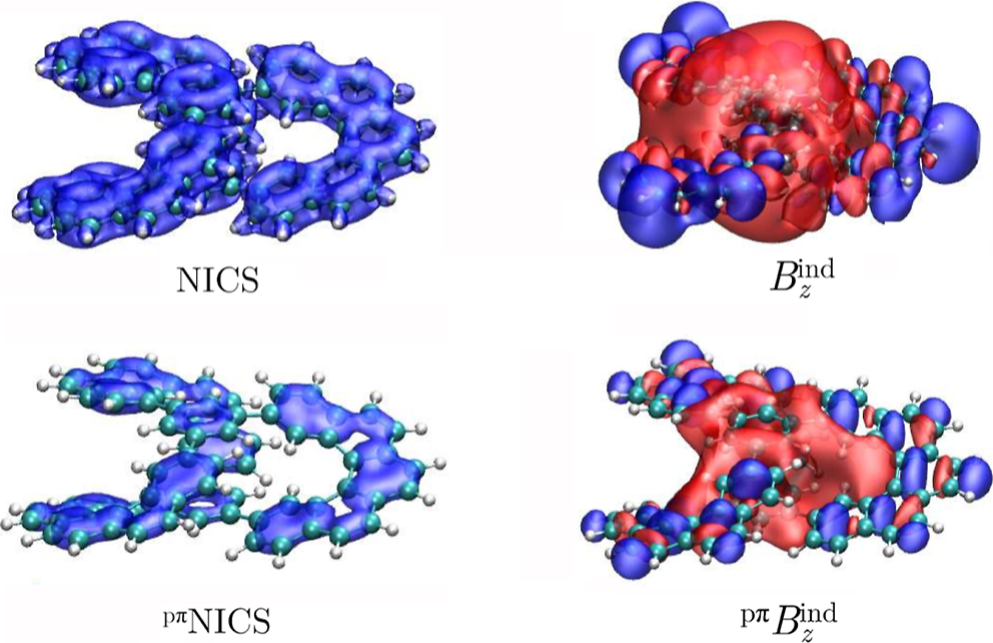
Isosurfaces of the total and the pseudo-π modeled NICS and *B*_*z*_^ind^ functions for **II**. The shielding (−15 ppm) and deshielding (+15 ppm) cones are shown in blue and red, respectively.

Current–density calculations elucidate that the magnetic shielding functions arise from a diatropic current density flowing along the periphery of the helicene branches (Figure 6 and animations in Supporting Information). Similar to the case of **I**, the helicene-like π currents are disconnected as in **I** and there are local current–density circulations in the stacked central 6-MRs. Integrating the current densities yields a ring-current strength of 8.25 (7.52) nA/T (pseudo-π) and 11.80 (12.67) nA/T for the central 6-MRs and helicene-like circuit, respectively (Table 1). Judging from the magnetic response, **I** and **II** have the same degree of aromaticity in their helicene branches, while the central 6-MRs in **II** are less aromatic than in **I**.

**Figure 6:**
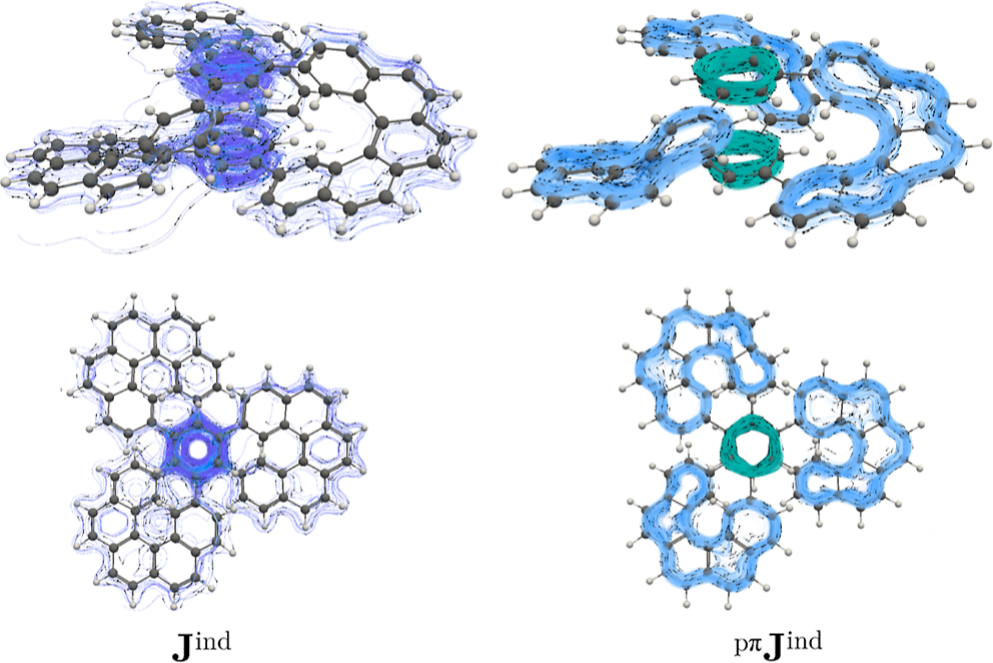
Streamlines and vector representation of the total and the pseudo-π modeled ring current density of **II**. Side view (top) and top view (bottom). The diatropic ring currents in the helicene branches and in the central 6-MR are shown in the ^pπ^ **J** ^ind^ in blue and green, respectively.

The electronic indices and EDDB-function calculations confirm the aromatic nature. Numerically, the total and the π-component of the EDDB functions support practically the same electron delocalization in the 6-MRs and the helicene branches of **II** as compared to those of **I**. The π-EDDB function also shows that the 6-MRs and 22-MRs have 75 and 65% π electron delocalization, respectively. Similarly, the C–C bridges connecting the 6-MRs with the helicene-arms yield DI values of 1.04, further suggesting that there is no global π-delocalization in molecule **II**, which agrees with the results obtained in the current density calculations.

Note that **I** and **II** exhibit a completely different electron-delocalization pattern as compared to [12]­infinitene, which exhibits a global aromatic character since it lacks the single-bond bridges of **I** and **II**. Since all 6-MRs of [12]­infinitene are fused, it can sustain global nonintersecting π-electron ring currents along its edges (see Figure S3). The EDDB plots of [12]­infinitene also display more continuous surfaces than for **I** and **II** (Figure S4). The global electronic indices computed for [12]­infinitene yielded values that are similar to the global values of **I** and **II** (Table S1), which can be interpreted as they are molecules with a similar but weak degree of aromaticity.

## Conclusions

The calculations elucidate that the [5]­helicene-based structures, denoted as **I** and **II**, exhibit local aromatic character. Due to the single-bond C–C connection of the helicene arms to the stacked central 6-MRs, the π delocalization is divided into local delocalized moieties consisting of the 6-MRs and the [5]­helicene branches. In the presence of an external magnetic field, molecules **I** and **II** sustain local diatropic ring currents around the 6-MRs and the helicene moieties. Integration of the ring-current strengths corroborates the local aromatic character of the 6-MRs, which are weaker than for benzene. The degree of aromaticity of the helicene branches is similar to that of benzene. However, it is challenging to determine current–density pathways and their aromatic nature based on magnetic shielding calculations. Even though the molecular structure of the studied molecules is reminiscent of the molecular structure of [12]­infinitene, the C–C single bonds between the 6-MRs and the helicene moieties prevent them from being globally aromatic.

Electronic aromaticity indices confirm that the stacked 6-MRs are less aromatic than benzene. The helicene moieties are even less aromatic than the 6-MRs. Calculations of the π-EDDB function and the DI indices also support the notion that the π delocalization is interrupted at the C–C single-bond connectors. The π-EDDB calculations also reveal that the percentage of π delocalized electrons in the 6-MR and in the helicene-like moieties is smaller than for benzene, resulting in a lower degree of aromaticity. The magnetic response calculations indicate a local aromatic character of the 22-MR units, which is comparable to that of benzene. The electronic indices suggest that the π delocalization of the helicene moieties is smaller than for benzene and the 6-MRs. The electronic indices also reveal that the 22-MRs are nonaromatic. Such contrasting conclusions between magnetic and electronic criteria of aromaticity often arise in studies of large rings, such as the helicene moieties. According to the employed electronic indices, the electron delocalization decreases with increasing size of the conjugated organic ring. The smaller electron delocalization obtained in the calculations of electronic indices does not prevent the molecules from sustaining a ring current when they are exposed to an external magnetic field. The present study shows that the same degree of aromaticity and aromatic nature are not always obtained when using magnetic criteria and electronic aromaticity indices. However, we showed that both electronic and magnetic criteria suggest that the studied molecules are not globally aromatic.

## Supplementary Material





## Data Availability

The data underlying this study are available in the published article and its Supporting Information.

## Notes

Animations of the current densities (ZIP)
Scheme of the integration planes, AV_min_ colored representation across a 6- and 22-MR circuit in **I** and **II**, the pseudo-π modeled current–density of [12]­infinitene, EDDB function plotted for [12]­infinitene, Electronic indices (BOA, AV1245, AV_min_) for [12]­infinitene, Cartesian coordinates of the optimized structures (PDF)
