# Supplementary figures and images for: Understanding Aromaticity in [5]Helicene-Bridged Cyclophanes: A Comprehensive Study
Source: J Org Chem. 2024 Jan 18;89(4):2459–66. doi: 10.1021/acs.joc.3c02485 (PMC12865772; doi:10.1021/acs.joc.3c02485)

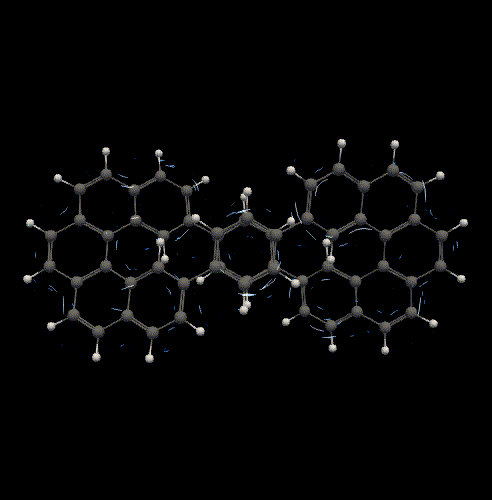

Supplement: Supplementary file 1 [file jo3c02485_si_001.zip › current-density-pseudo-pi-I_system.gif]

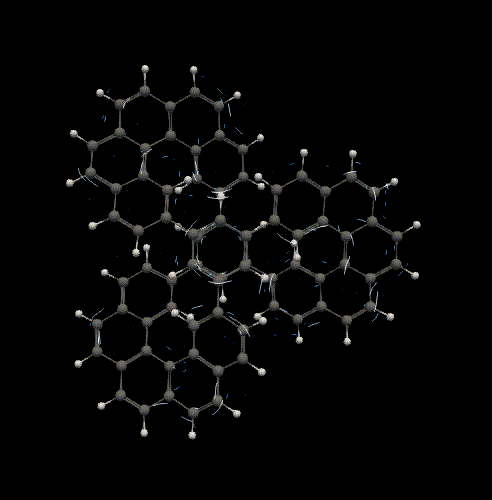

Supplement: Supplementary file 1 [file jo3c02485_si_001.zip › current-density-pseudo-pi-II_system.gif]

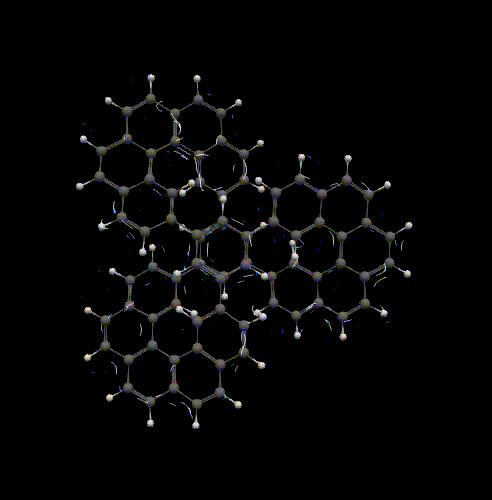

Supplement: Supplementary file 1 [file jo3c02485_si_001.zip › current-density-total-II_system.gif]

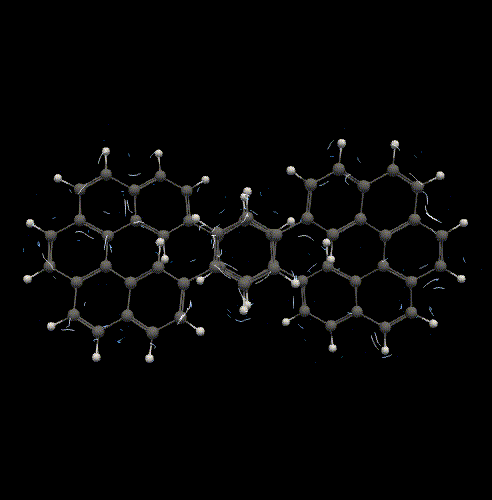

Supplement: Supplementary file 1 [file jo3c02485_si_001.zip › curret-density-total-I_system.gif]
