# Supplementary material for: Understanding Aromaticity in [5]Helicene-Bridged Cyclophanes: A Comprehensive Study
Source: J Org Chem. 2024 Jan 18;89(4):2459–66. doi: 10.1021/acs.joc.3c02485 (PMC12865772; doi:10.1021/acs.joc.3c02485)
Supplement: Supplementary file 2 [file jo3c02485_si_002.pdf]

Supporting Information:
Understanding Aromaticity in [5]Helicene-Bridged Cyclophanes: A Comprehensive Study

Mesías Orozco-Ic,[1,*] Luis Soriano-Agueda,[1] Sílvia Escayola,[1] Dage Sundholm,[3] Gabriel Merino,[4] Eduard Matito.[1]

1 *Donostia International Physics Center (DIPC), 20018 Donostia, Euskadi, Spain.*
2 *Institut de Química Computacional i Catàlisi and Departament de Química, Universitat de Girona, C/ Maria Aurèlia Capmany, 69, Girona, Catalonia, 17003, Spain.*
3 *Department of Chemistry, Faculty of Science, University of Helsinki, P.O. Box 55, A. I. Virtasen aukio 1, FIN-00014 Helsinki, Finland.*
4 *Departamento de Física Aplicada, Centro de Investigación y de Estudios Avanzados, Unidad Mérida. Km 6 Antigua Carretera a Progreso. Apdo. Postal 73, Cordemex, 97310, Mérida, Yuc., México.*

E-mail: mesias.orozco@dipc.org

Table of contents

Figure S1. Scheme of the integration planes in the studied structures used to compute the ring-current strengths in Table 1 .... S2

Figure S2. AV_{min} colored representation across a 6- and 22-MR circuit in **I** and **II**. Green, orange, and red colors indicate bond contributions to conjugation that are, respectively, 75−100%, 5−75%, and 0−25%. S2

Figure S3. Streamlines and vector representation of the pseudo-π modeled current-density of infinitene. The diatropic global non-intersecting ring currents are displayed in purple and green, respectively. See *Phys. Chem. Chem. Phys.*, 2022, 24, 6404-6409 and *J. Phys. Chem. A*, 2022, 126, 3717-3723 for more details. S3

Figure S4. The total and π-contributions to the EDDB function plotted for [12]infinitene. The plotted isosurfaces correspond to an isovalue of 0.02. .... S3

Table S1. Electronic indices (BOA, AV1245, AVmin) for the global structure of [12]infinitene, computed at the CAM-B3LYP/def2-TZVP level. S4

Cartesian coordinates of the optimized structures computed at the CAM-B3LYP-D3(BJ)/def2-TZVP level. S4

The animations of the total and pseudo-π magnetically induced current densities of **I** and **II** are

provided as separated *.gif files.

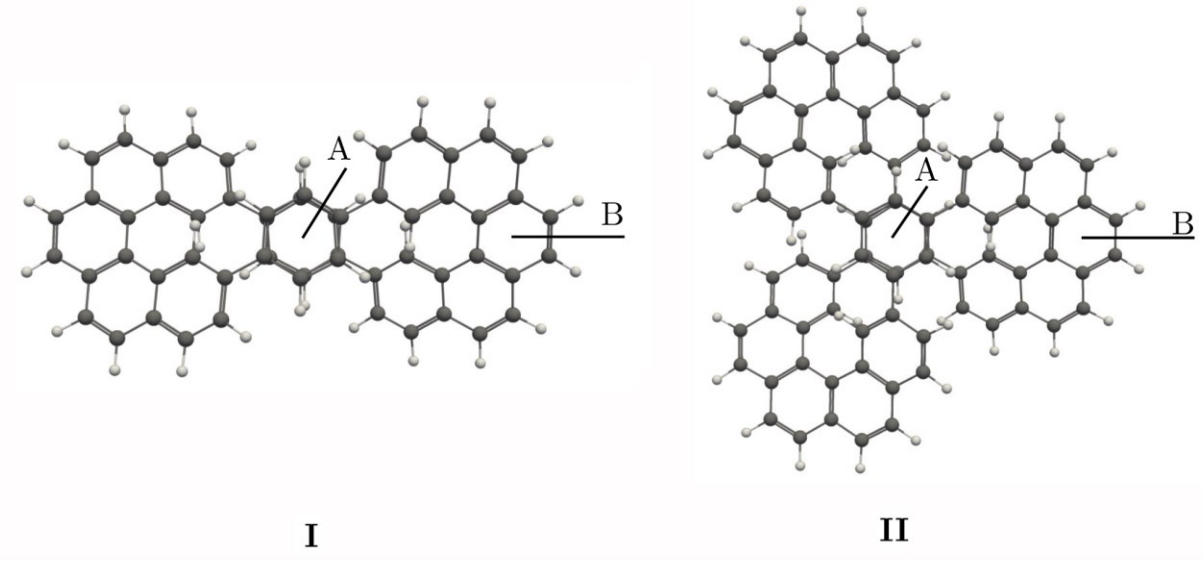

Figure S1. Scheme of the integration planes in the studied structures used to compute the ring-current strengths in Table 1.

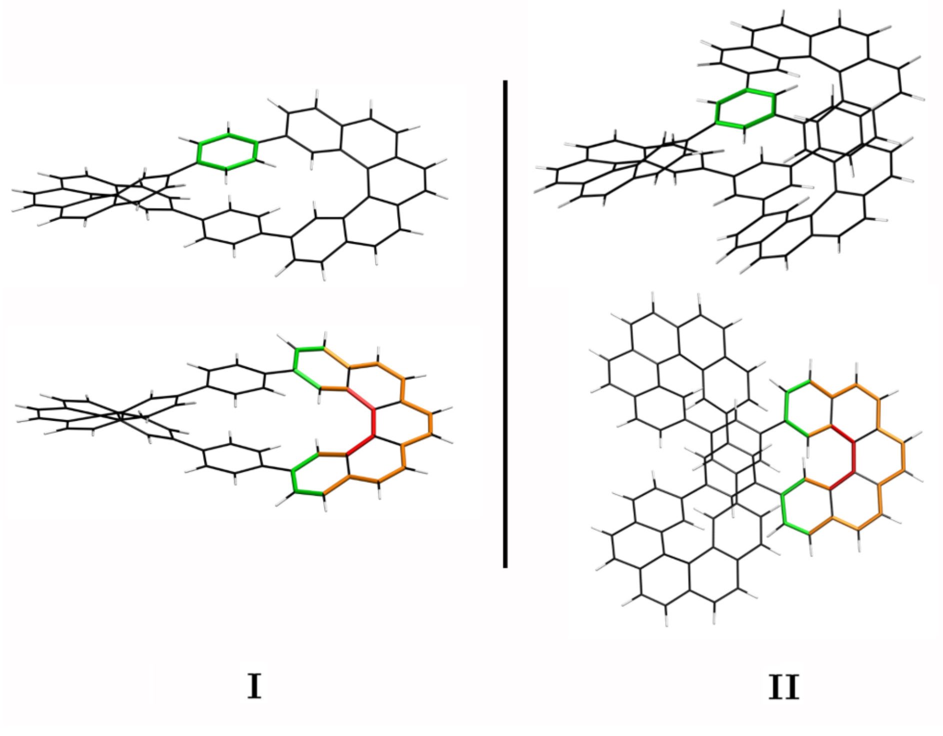

Figure S2. AV_{min} colored representation across a 6- and 22-MR circuit in **I** and **II**. Green, orange, and red colors indicate bond contributions to conjugation that are, respectively, 75−100%, 5−75%, and 0−25%.

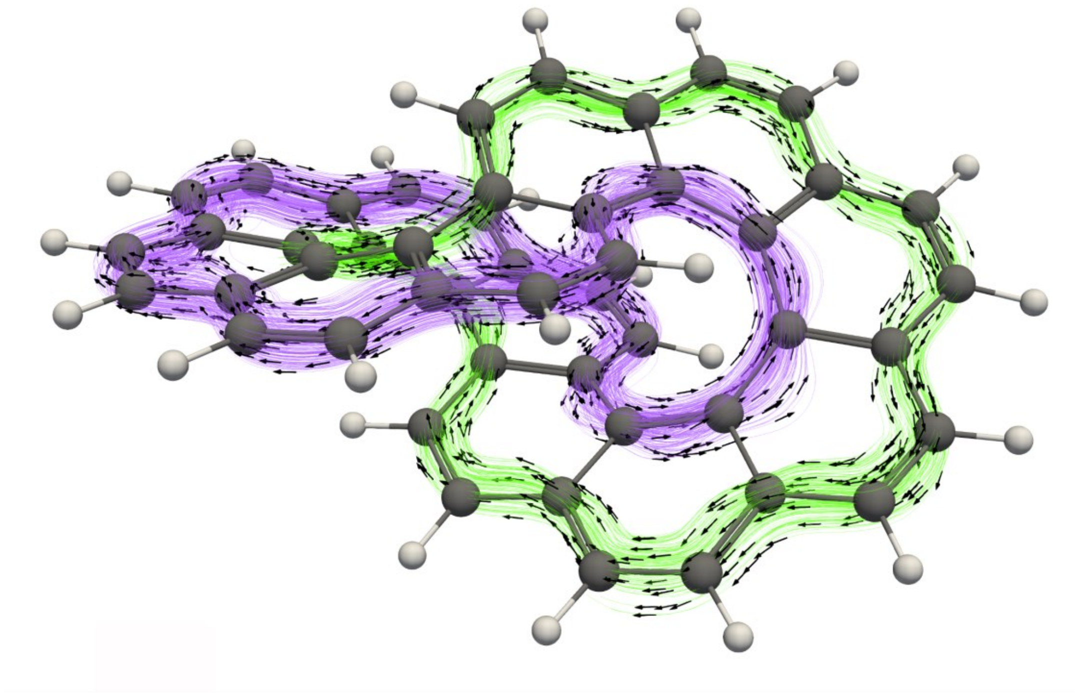

Figure S3. Streamlines and vector representation of the pseudo-π modeled current-density of [12]infinitene. The diatropic global non-intersecting ring currents are displayed in purple and green, respectively. See *Phys. Chem. Chem. Phys.*, 2022, 24, 6404-6409 and *J. Phys. Chem. A*, 2022, 126, 3717-3723 for more details.

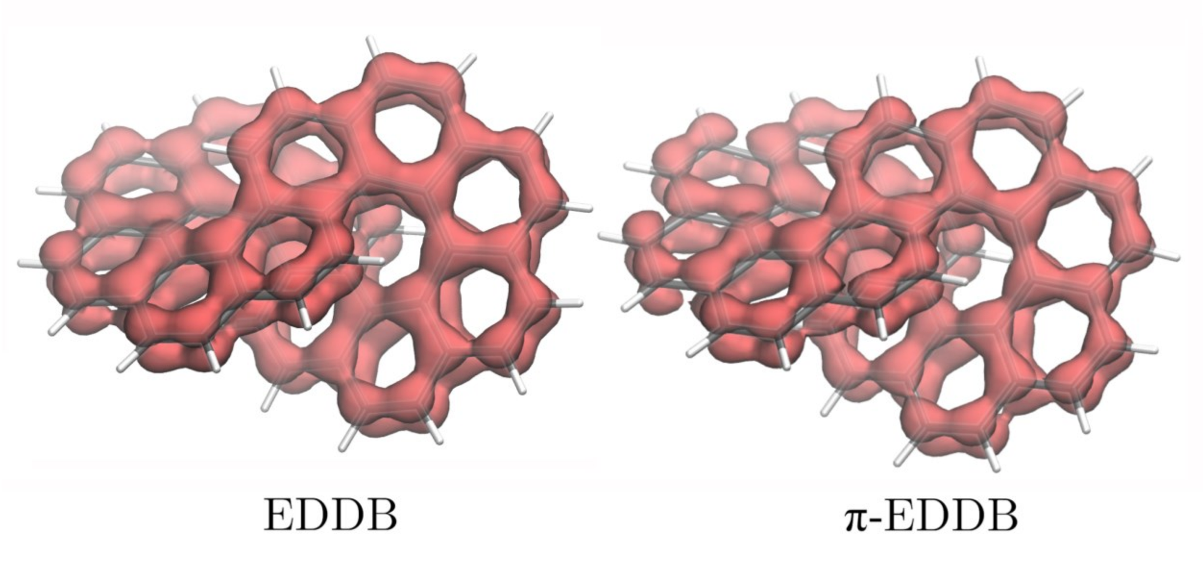

Figure S4. The total and π-contributions to the EDDB function plotted for [12]infinitene. The plotted isosurfaces correspond to an isovalue of 0.02.

Table S1. Electronic indices (BOA, AV1245, AVmin) for the global structure of [12]infinitene, computed at the CAM-B3LYP/def2-TZVP level.

Molecule	Ring	BOA	AV1245	AV_{min}
[12]infinitene	Global	0.2266	1.498	0.115

Cartesian coordinates of the optimized structures computed at the CAM-B3LYP-D3(BJ)/def2-TZVP level.

I

C	1.258411100	0.653352900	1.899885200
C	8.448810100	0.660166500	0.136596000
C	5.997549200	0.662115400	0.296373100
C	7.230528800	1.337937600	0.376047600
C	7.296036600	2.713968200	0.739782600
C	6.193675800	3.394106300	1.099731300
C	3.656581900	0.690172700	1.243800000
C	4.854309200	1.340964800	0.896685600
C	4.957060800	2.709856400	1.234698200
C	3.845958700	3.374274000	1.783011200
C	2.671859800	2.725984700	2.013490100
C	2.555026400	1.348917600	1.742277600
C	2.555026400	-1.348917600	-1.742277600
C	2.671859800	-2.725984700	-2.013490100
C	3.845958700	-3.374274000	-1.783011200
C	4.957060800	-2.709856400	-1.234698200
C	4.854309200	-1.340964800	-0.896685600
C	3.656581900	-0.690172700	-1.243800000
C	6.193675800	-3.394106300	-1.099731300
C	7.296036600	-2.713968200	-0.739782600
C	7.230528800	-1.337937600	-0.376047600
C	5.997549200	-0.662115400	-0.296373100
C	8.448810100	-0.660166500	-0.136596000
C	1.258411100	-0.653352900	-1.899885200
C	0.062678900	-1.368129600	-1.919820300
C	-1.161448200	-0.734264500	-1.929327300
C	-1.258411100	0.653352900	-1.899885200
C	-0.062678900	1.368129600	-1.919820300
C	1.161448200	0.734264500	-1.929327300
C	0.062678900	1.368129600	1.919820300
C	-1.161448200	0.734264500	1.929327300

C	-1.258411100	-0.653352900	1.899885200
C	-0.062678900	-1.368129600	1.919820300
C	1.161448200	-0.734264500	1.929327300
C	-8.448810100	-0.660166500	0.136596000
C	-5.997549200	-0.662115400	0.296373100
C	-7.230528800	-1.337937600	0.376047600
C	-7.296036600	-2.713968200	0.739782600
C	-6.193675800	-3.394106300	1.099731300
C	-3.656581900	-0.690172700	1.243800000
C	-4.854309200	-1.340964800	0.896685600
C	-4.957060800	-2.709856400	1.234698200
C	-3.845958700	-3.374274000	1.783011200
C	-2.671859800	-2.725984700	2.013490100
C	-2.555026400	-1.348917600	1.742277600
C	-2.555026400	1.348917600	-1.742277600
C	-2.671859800	2.725984700	-2.013490100
C	-3.845958700	3.374274000	-1.783011200
C	-4.957060800	2.709856400	-1.234698200
C	-4.854309200	1.340964800	-0.896685600
C	-3.656581900	0.690172700	-1.243800000
C	-6.193675800	3.394106300	-1.099731300
C	-7.296036600	2.713968200	-0.739782600
C	-7.230528800	1.337937600	-0.376047600
C	-5.997549200	0.662115400	-0.296373100
C	-8.448810100	0.660166500	-0.136596000
H	9.375208200	1.210315300	0.237195300
H	8.263492100	3.198477300	0.713192000
H	6.240798800	4.444921900	1.354225100
H	3.587419600	-0.368247100	1.073879200
H	3.947725800	4.421882600	2.036935000
H	1.842055100	3.268422500	2.441275800
H	1.842055100	-3.268422500	-2.441275800
H	3.947725800	-4.421882600	-2.036935000
H	3.587419600	0.368247100	-1.073879200
H	6.240798800	-4.444921900	-1.354225100
H	8.263492100	-3.198477300	-0.713192000
H	9.375208200	-1.210315300	-0.237195300
H	0.075962900	-2.447272700	-1.881958800
H	-2.054287500	-1.342566700	-1.931100000
H	-0.075962900	2.447272700	-1.881958800
H	2.054287500	1.342566700	-1.931100000
H	0.075962900	2.447272700	1.881958800
H	-2.054287500	1.342566700	1.931100000
H	-0.075962900	-2.447272700	1.881958800

H	2.054287500	-1.342566700	1.931100000
H	-9.375208200	-1.210315300	0.237195300
H	-8.263492100	-3.198477300	0.713192000
H	-6.240798800	-4.444921900	1.354225100
H	-3.587419600	0.368247100	1.073879200
H	-3.947725800	-4.421882600	2.036935000
H	-1.842055100	-3.268422500	2.441275800
H	-1.842055100	3.268422500	-2.441275800
H	-3.947725800	4.421882600	-2.036935000
H	-3.587419600	-0.368247100	-1.073879200
H	-6.240798800	4.444921900	-1.354225100
H	-8.263492100	3.198477300	-0.713192000
H	-9.375208200	1.210315300	-0.237195300

II

C	3.639067600	0.714640800	1.219483400
H	3.593462300	-0.350689400	1.097366700
C	7.204891900	1.351812100	0.331067000
C	4.828069700	-1.364854000	-0.846011500
C	5.974600600	-0.670982500	-0.273397900
C	-1.192975500	-0.735460100	-1.853360200
C	2.525043400	1.377812400	1.680271400
C	7.260903900	-2.741312500	-0.644633400
C	-3.596033100	-3.498804000	-0.846011500
C	4.916809200	-2.746511700	-1.124858500
C	1.233414900	-0.665417100	-1.853360200
C	4.828069700	1.364854000	0.846011500
C	-3.568388200	-4.838664600	-0.273397900
C	6.152066900	-3.430410600	-0.969345600
C	-2.406212400	-5.509647100	0.273397900
C	-2.455742300	-1.497845500	-1.680271400
C	-6.046856200	-3.612640900	-0.969345600
C	0.041434600	-1.375308800	-1.863056400
H	0.074091400	-2.450326800	-1.815801600
C	-6.004498200	-4.917471000	-0.644633400
C	1.233414900	0.665417100	1.853360200
C	7.260903900	2.741312500	0.644633400
C	-1.232036600	-4.863658000	0.846011500
C	1.170335000	0.723537800	-1.863056400
H	2.084999600	1.289328500	-1.815801600
C	-4.836953500	-2.884825900	-1.124858500
C	2.525043400	-1.377812400	-1.680271400
C	7.204891900	-1.351812100	-0.331067000
C	1.086139400	-3.655904600	1.881952500

H	1.989077800	-3.207671800	2.267994900
C	-0.079855700	-5.631337500	1.124858500
C	3.639067600	-0.714640800	-1.219483400
H	3.593462300	0.350689400	-1.097366700
C	-0.040439300	1.400877100	-1.853360200
C	-4.773149600	-5.563713400	-0.331067000
C	6.152066900	3.430410600	0.969345600
C	-0.069301100	-2.875657900	1.680271400
C	-0.040439300	-1.400877100	1.853360200
C	8.422873700	0.664744100	0.115321100
H	9.349978500	1.216621900	0.198305900
C	-1.256405700	-7.658783500	0.644633400
C	5.974600600	0.670982500	0.273397900
C	-1.211769700	0.651770900	-1.863056400
H	-2.159091000	1.160998400	-1.815801600
C	-2.438430900	-2.794204600	-1.219483400
H	-1.493025200	-3.287374300	-1.097366700
C	1.170335000	-0.723537800	1.863056400
H	2.084999600	-1.289328500	1.815801600
C	2.623036600	-2.768576600	-1.881952500
H	1.783386400	-3.326427800	-2.267994900
C	-1.200636700	-3.508845400	1.219483400
H	-2.100437100	-2.936684900	1.097366700
C	-0.105210700	-7.043051500	0.969345600
C	-4.787122100	-6.962050600	-0.115321100
H	-5.728614700	-7.489008000	-0.198305900
C	-2.431742300	-6.915525500	0.331067000
C	-1.192975500	0.735460100	1.853360200
C	4.916809200	2.746511700	1.124858500
C	3.791891000	-3.420722600	-1.632066400
H	3.874433200	-4.480780200	-1.835259800
C	-4.858378100	-1.573512600	-1.632066400
H	-5.817686100	-1.114967500	-1.835259800
C	-0.069301100	2.875657900	-1.680271400
C	-3.635751600	-7.626794700	0.115321100
H	-3.621363800	-8.705629900	0.198305900
C	8.422873700	-0.664744100	-0.115321100
H	9.349978500	-1.216621900	-0.198305900
C	0.041434600	1.375308800	1.863056400
H	0.074091400	2.450326800	1.815801600
C	1.066487100	-4.994235200	1.632066400
H	1.943252900	-5.595747700	1.835259800
C	-3.709176000	-0.887328000	-1.881952500
H	-3.772464100	0.118756000	-2.267994900

C	-1.211769700	-0.651770900	1.863056400
H	-2.159091000	-1.160998400	1.815801600
C	3.791891000	3.420722600	1.632066400
H	3.874433200	4.480780200	1.835259800
C	-2.455742300	1.497845500	1.680271400
C	2.623036600	2.768576600	1.881952500
H	1.783386400	3.326427800	2.267994900
C	-1.200636700	3.508845400	-1.219483400
H	-2.100437100	2.936684900	-1.097366700
C	-0.079855700	5.631337500	-1.124858500
C	1.086139400	3.655904600	-1.881952500
H	1.989077800	3.207671800	-2.267994900
C	-2.438430900	2.794204600	1.219483400
H	-1.493025200	3.287374300	1.097366700
C	1.066487100	4.994235200	-1.632066400
H	1.943252900	5.595747700	-1.835259800
C	-3.596033100	3.498804000	0.846011500
C	-1.232036600	4.863658000	-0.846011500
C	-3.709176000	0.887328000	1.881952500
H	-3.772464100	-0.118756000	2.267994900
C	-3.568388200	4.838664600	0.273397900
C	-4.836953500	2.884825900	1.124858500
C	-2.406212400	5.509647100	-0.273397900
C	-4.858378100	1.573512600	1.632066400
H	-5.817686100	1.114967500	1.835259800
C	-0.105210700	7.043051500	-0.969345600
C	-2.431742300	6.915525500	-0.331067000
C	-1.256405700	7.658783500	-0.644633400
C	-4.773149600	5.563713400	0.331067000
C	-4.787122100	6.962050600	0.115321100
H	-5.728614700	7.489008000	0.198305900
C	-3.635751600	7.626794700	-0.115321100
H	-3.621363800	8.705629900	-0.198305900
C	-6.004498200	4.917471000	0.644633400
C	-6.046856200	3.612640900	0.969345600
H	-6.910236900	-5.508722200	-0.605656200
H	-6.985705500	-3.117776200	-1.181372600
H	-1.315574900	-8.738801800	0.605656200
H	0.792779400	-7.608686600	1.181372600
H	6.192926200	-4.490910400	-1.181372600
H	8.225811800	-3.230079600	-0.605656200
H	8.225811800	3.230079600	0.605656200
H	6.192926200	4.490910400	1.181372600
H	0.792779400	7.608686600	-1.181372600

H	-1.315574900	8.738801800	-0.605656200
H	-6.910236900	5.508722200	0.605656200
H	-6.985705500	3.117776200	1.181372600
